# Assessing the Pilot Implementation of the Integrated Multimorbidity Care Model in Five European Settings: Results from the Joint Action CHRODIS-PLUS

**DOI:** 10.3390/ijerph17155268

**Published:** 2020-07-22

**Authors:** Carmen Rodriguez-Blazquez, Maria João Forjaz, Antonio Gimeno-Miguel, Kevin Bliek-Bueno, Beatriz Poblador-Plou, Sara Pilar Luengo-Broto, Inmaculada Guerrero-Fernández de Alba, Ana Maria Carriazo, Carmen Lama, Rafael Rodríguez-Acuña, Inmaculada Cosano, Juan José Bedoya, Carmen Angioletti, Angelo Carfì, Antonella Di Paola, Rokas Navickas, Elena Jureviciene, Laimis Dambrauskas, Ida Liseckiene, Leonas Valius, Gediminas Urbonas, Graziano Onder, Alexandra Prados-Torres

**Affiliations:** 1 National Centre of Epidemiology, Institute of Health Carlos III and CIBERNED, 28029 Madrid, Spain; crodb@isciii.es; 2 National Centre of Epidemiology, Institute of Health Carlos III and REDISSEC, 28029 Madrid, Spain; 3 EpiChron Research Group, Aragon Health Sciences Institute (IACS), IIS Aragon, REDISSEC, Miguel Servet University Hospital, 50009 Zaragoza, Spain; agimenomi.iacs@aragon.es (A.G.-M.); bpoblador.iacs@aragon.es (B.P.-P.); sprados.iacs@aragon.es (A.P.-T.); 4 Teaching Unit of Preventive Medicine and Public Health, Miguel Servet University Hospital, 50009 Zaragoza, Spain; kbliek@salud.aragon.es (K.B.-B.); saraluengoria@gmail.com (S.P.L.-B.); inmagfa@gmail.com (I.G.-F.d.A.); 5 Regional Ministry of Health and Families of Andalusia, E-41020 Seville, Spain; anam.carriazo@juntadeandalucia.es (A.M.C.); carmenm.lama@juntadeandalucia.es (C.L.); 6 Andalusian Public Foundation Progress and Health (FPS), E-41092 Seville, Spain; rafael.rodriguez.acuna@juntadeandalucia.es; 7 Servicio Andaluz de Salud (SAS), San Jose de la Rinconada-Los Carteros Primary Care Center, E-41300 Seville, Spain; inmaculada.cosano.sspa@juntadeandalucia.es; 8 Servicio Andaluz de Salud (SAS), Tiro de Pichon Primary Care Center, E-29006 Malaga, Spain; bedoyabelmonte@gmail.com; 9 Department of Internal Medicine and Geriatrics, Universita Cattolica del Sacro Cuore (UCSC), 00168 Rome, Italy; carmen.angioletti@unicatt.it (C.A.); antonella.dipaola@unicatt.it (A.D.P.); 10 Centro di Medicina dell’Invecchiamento, Fondazione Policlinico Universitario A. Gemelli IRCCS, 00168 Rome, Italy; angelo.carfi@policlinicogemelli.it; 11 Faculty of Medicine, Vilnius University, LT-03101 Vilnius, Lithuania; Rokas.Navickas@santa.lt (R.N.); Elena.Jureviciene@santa.lt (E.J.); Laimis.Dambrauskas@santa.lt (L.D.); 12 Department of Biomedical Research, Vilnius University Hospital Santaros Klinikos, LT-08661 Vilnius, Lithuania; 13 Family Medicine Clinic, Hospital of Lithuanian University of Health Sciences Kauno Klinikos, 50161 Kaunas, Lithuania; ida.liseckiene@kaunoklinikos.lt (I.L.); seimos.medicinos.klinika@kaunoklinikos.lt (L.V.); gediminas.urbonas@fc.lsmuni.lt (G.U.); 14 Department of Family Medicine, Lithuanian University of Health Sciences, 44307 Kaunas, Lithuania; 15 Department of Cardiovascular, Endocrine-Metabolic Diseases and Aging, Istituto Superiore di Sanita, 0161 Rome, Italy; graziano.onder@iss.it

**Keywords:** multimorbidity, integrated multimorbidity care model, chronic diseases, implementation research, non-communicable diseases, integrated care, care model, individualized care plans, comprehensive assessment

## Abstract

Multimorbidity, the coexistence of several chronic conditions in a patient, represents a great challenge for healthcare systems and society. The Integrated Multimorbidity Care Model (IMCM) was recently designed within the Joint Action on chronic diseases and promoting healthy ageing across the life cycle (CHRODIS) to ensure the continuity of care for patients with multimorbidity. The IMCM was implemented in five European pilot sites in Spain, Italy, and Lithuania, within the Joint Action CHRODIS-PLUS. The effect of these pilot interventions was assessed pre- and post-implementation by 17 healthcare managers, using the Assessment of Chronic Illness Care (ACIC) measure, and by 226 patients with the Patient Assessment of Care for Chronic Conditions (PACIC+) survey. The ACIC total score significantly increased (5.23 to 6.71, *p* = 0.022) after the intervention, with differences across sites. A significant increase in the PACIC+ summary score was found ranging from 3.25 at baseline to 4.03 after the intervention (*p* < 0.001), and 58% of the sample perceived an improvement in care. Higher PACIC+ scores after the intervention were associated to lower baseline values in the respective PACIC+ dimension and to greater changes in ACIC Part 1 (delivery system organization). The IMCM implementation can help improve the quality of care for patients with multimorbidity.

## 1. Introduction

Multimorbidity, defined as the coexistence of several chronic conditions in the same individual, has become the norm in older adults in high-income countries, affecting more than 60% of people aged 65 or over and, therefore, representing a growing global health concern [1,2]. Multimorbidity affects patients and their families, as it is associated to greater disability risk, lower quality of life, and premature death of patients [3], as well as to reduced quality of life of patients’ family members and caregivers. It also poses a challenge for overworked clinicians and a financial burden for health and social care systems [4].

Several interventions and models of care for patients with multimorbidity have been developed during the past years to address this problem, such as the patient-centered 3D approach [5], the Ariadne principles [6], the MULTIPAP intervention [7], and the NICE guideline for the clinical management of multimorbidity [8] to cite just a few. However, most of the models currently available have been developed within clinical trials and have not been implemented in real-life conditions, or specifically focus health care aspects without considering other relevant dimensions such as social and community resources, therefore not providing comprehensive frameworks adaptable to different health care scenarios.

In this context, the Joint Action on chronic diseases and promoting healthy ageing across the life cycle (CHRODIS) recently developed the Integrated Multimorbidity Care Model (IMCM), based on the Chronic Care Model [9] and the Innovative Care for Chronic Conditions Model [10]. The IMCM includes sixteen components across five domains (i.e., care delivery, decision and self-management support, technology, and community/social resources) [11]. This model was not developed to be fully implemented as a whole but to serve as a flexible model to be adapted to national and/or local peculiarities, so that each implementing site can select and assess which dimension/s to implement and how. The potential applicability of the IMCM has been already assessed through a theoretical case study of an older adult with diabetes and mental health diseases showing a potential [12].

This initiative continued with CHRODIS-PLUS (implementing good practices for chronic diseases), a three-year (2017–2020) Joint Action coordinated by the European Commission aimed at piloting the implementation of the IMCM in different health care settings in Europe, among other objectives. The common implementation methodology that was followed, as well as the baseline characteristics, specific aims, and action plans of each of the five implementing sites selected in Italy, Lithuania, and Spain has been published elsewhere [13]. One of the main objectives of CHRODIS-PLUS was to assess the success and impact of the IMCM pilot implementation, and to provide local adaptations of the model based on the results obtained in each pilot site. For this, the perspectives of the patient and the health system stakeholders regarding the quality of provided care were agreed upon as key common indicators.

The aim of this work is to analyze and assess the impact of the pilot IMCM intervention across the five implementing sites by comparing specific indicators measured pre- and post- implementation.

## 2. Materials and Methods

### 2.1. Study Design and Participants

This is a pilot, descriptive study, with pre- and post- implementation assessments, designed to evaluate the impact of the IMCM in five pilot healthcare implementation sites from both the primary and specialized level from three European countries. The participating sites were: Andalusian Department of Health in Andalusia, and Aragon Department of Health in Aragon, both in Spain; Gemelli Hospital, Catholic University of the Sacred Heart in Rome, Italy; Hospital of the Lithuanian University of Health Sciences Kauno Klinikos in Kaunas, and Vilnius University Hospital Santaros Klinikos in Vilnius, both in Lithuania. A description of the characteristics of each national healthcare system and implementation site has been previously published [13]. From this point on and for word-economy purposes, the geographical name will be used when referring to each implementation site. For instance, when referring to the Rome implementation site of Gemelli Hospital, Catholic University of the Sacred Heart, Rome will be used instead. However, our results are not generalizable to the whole city or region.

The main inclusion criterion for patients was having multimorbidity, defined as the presence of two or more chronic conditions, although specific inclusion criteria differed by site. Specific inclusion criteria by site are described in the next section.

### 2.2. IMCM Interventions

The implementation of the IMCM followed a structured methodology, common to each participating site, but specifically adapted to their specific characteristics, based on baseline situation analyses conducted before the intervention [13]. The duration of the intervention was 12 months, and assessments were performed at the beginning and end of the intervention. All interventions were integrated as standard practice of the respective sites.

The team in Andalusia integrated the implementation within the framework of existing initiatives for complex chronic patients in primary care [14]. The Andalusian intervention targeted individualized care plans with a focus on the improvement of management of complex chronic patients (patients with chronic severe health problems, multimorbidity, and polypharmacy) while other components of the IMCM had already been incorporated into the routine clinical practice in Andalusia. Individualized comprehensive care plans were therefore systematically drafted and delivered to complex chronic patients with no age limits (n = 2788) and were subsequently followed for one year [14,15,16,17].

The implementation in Aragon, Spain, was also designed within the framework of the existing program for complex chronic patients of the Aragon Health System and included both primary and specialized care centers. This intervention specifically comprised the training of health care professionals in multimorbidity through the online course eMULTIPAP based on the Ariadne principles for the management of multimorbidity in primary care from a patient-centered perspective [7]. The local team targeted components from all five IMCM domains and included 291patients with multimorbidity (≥3 chronic diseases) and polypharmacy (≥5 drugs) aged 65 years and over. In addition to the online course, the intervention included appointing case managers, creating individualized care plans, promoting community resource use, and developing a virtual consultation system [13].

The setting for the implementation in Rome, Italy was a day hospital from a tertiary care center and the implementation focused on adults with dementia (n = 17) and with intellectual disability aged 65 years or older (n = 248). The program targeted components from all five IMCM domains and was orientated towards the coordination, accessibility, and integration of care, prioritizing self-management and career development through patient-operated technology, and encouraging multidisciplinary collaborations [13].

The Lithuanian sites, Kaunas and Vilnius, targeted components from all five domains of the model and included primary and specialized care professionals at the implementation, which was directed at 201 and 195 patients with multimorbidity, respectively, aged 40 years and older. Both site teams aimed at optimizing healthcare resources and decreasing care fragmentation by appointing case managers and creating individualized care plans, developing consultation systems for professionals, and improving patient access to community resources [13]. Each pilot site from Lithuania adapted the IMCM according to the local context: one site, VULSK, prioritizing heavy users; the other, Kaunas, comparing the care delivery between rural and urban areas.

### 2.3. Measures

The perspectives of the patient and the health system regarding the quality of provided care were agreed upon as key common indicators for all implementing sites. Self-perceived patient care was assessed through the 26-item Patient Assessment of Care for Chronic Conditions (PACIC+) survey [18], which measures specific actions or qualities of care that patients report to have experienced during their interactions with the delivery system. The perspective of the health system teams was analyzed by using the Assessment of Chronic Illness Care (ACIC) survey [19], a practical quality-improvement tool to help organizations evaluate the strengths and weaknesses of their care delivery for chronic illnesses. Both questionnaires were collected and analyzed before and after the one-year model implementation.

An ad-hoc questionnaire was also developed to collect basic sociodemographic data of patients (i.e., sex, age, civil status, education level, and employment status). In addition, a specific question on perceived change (change score) was included after the implementation, asking “concerning your chronic conditions, please rate the degree of change in the care you have received in the past 12 months”. This item presented seven Likert-type response options, ranging from 1 (very much worse) to 7 (very much improved). Due to administrative issues, Rome did not provide ratings in the change score.

The ACIC (version 3.5) [19] assesses the strengths and weaknesses of delivery of care for chronic illness in seven areas: Delivery system organization (Part 1), community linkages (Part 2), self-management support (Part 3a), decision support (Part 3b), delivery system design (Part 3c), clinical information systems (Part 3d), and integration of model components (Part 4). The ACIC was applied in each site pre- and post-implementation. Items are scored from 0 (the lowest level of support) to 11 (the optimal level of support). Scores for each section are obtained by summing the values for all items within a section and dividing by the number of items within that section (range: 0–11). The overall score is derived by summing the average scores of each section and dividing by the number of sections administered (range: 0–11). The following ranges for quality of care levels have been established: 0–2 for limited support for multimorbidity care; 3–5 for basic support for multimorbidity care; 6–8 for reasonably good support for multimorbidity care; and 9–11 for fully developed support for multimorbidity care [20]. The ACIC was completed by members of the implementation team with a good knowledge on the implementation as well as site and health care system characteristics (decision maker, front-line stakeholder or implementer). The ACIC was discussed in a small group of people and the consensus was filled by one of the team members providing a joint position from several team members. The ACIC is responsive to changes that care teams make in their healthcare systems and correlates well with other measures of productivity and system change.

The PACIC+ [18] was selected for quantitative outcome assessment of the interventions as perceived by the patients. PACIC+ consists of 26 items, scored from 1 (almost never) to 5 (almost always). The PACIC+ allows for a scoring method derived from the “5As” model of behavioral counseling that defines five measurable outcomes: assess, advise, agree, assist, and arrange [21]. These dimensions permit measuring the improvement in self-management support and linkages to community resources [22]. A global “5As” summary score was also calculated, resulting from the average of items 1–4 and 6–16. The PACIC+ has been translated into several languages, thus Lithuanian, Italian, and Spanish versions were used.

### 2.4. Data Analysis

Descriptive statistics (frequencies and percentages, means and standard deviations) were used to characterize the participants and to summarize the results of ACIC and PACIC+ questionnaires, total and subscales scores, for each site. The ACIC scores were averaged by site and time (pre- or post-intervention). Pre-intervention PACIC+ data for Aragon was imputed, using the multiple regression model based on patient’s sociodemographic characteristics and the change score. This model explained 54.1% of the variance, and the correlation between the imputed and real data points (complete data set) was r = 0.74 (*p* < 0.001).

Differences in ACIC and PACIC+ scores between pre- and post-implementation assessments in each site were ascertained using the Mann-Whitney test for the ACIC and the Student’s paired *t*-test for the PACIC+. The magnitude of change was estimated using Cohen’s d formula for effect size (mean_t2_–mean_t1_)/SD_t1_) [23], with 0.50–0.79 indicating moderate change and ≥0.80, large change.

Six linear regression models, with the difference between post- and pre-implementation in all PACIC+ dimensions and total as dependent variables, respectively, were performed. As independent variables, the following were included in the models: sex, age, civil status (in two categories: with and without partner), education (two categories: primary or lower and secondary or higher), employment status (inactive and active), the respective baseline PACIC+ scores, and difference in ACIC dimensions 1, 2, and 4 scores. ACIC dimension 3 was not included due to strong collinearity with other dimensions.

Calculations were performed using the IBM SPSS, version 22.0, software, New York, United States.

## 3. Results

### 3.1. ACIC Results

Before the implementation, members of the implementation teams completed a total of 14 ACIC surveys (two from Andalusia, three from Aragon, two from Rome, two from Kaunas, and five from Vilnius). After the implementation, 17 ACIC surveys were completed (five from Andalusia, three from Aragon, two from Rome, two from Kaunas, and five from Vilnius).

**Figure 1:**
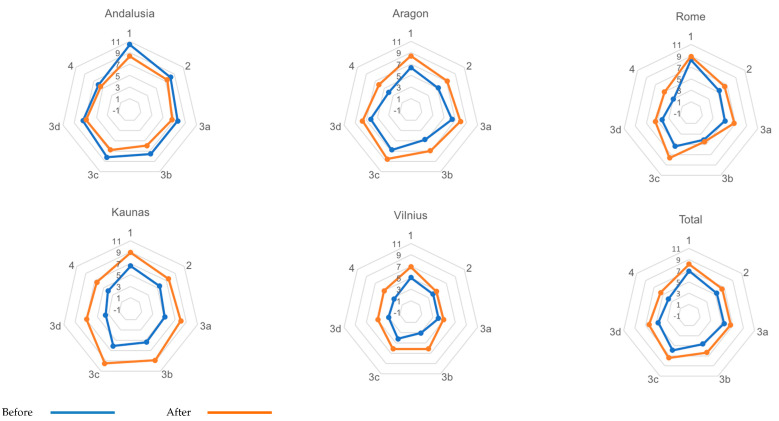
The ACIC mean scores before (pre) and after (post) implementation of the Integrated Multimorbidity Care Model (IMCM), by site and total sample. 1. Delivery system organization; 2. Community linkages; 3a. Self-management support; 3b. Decision support; 3c. Delivery system design; 3d. Clinical information systems; 4. IMCM component integration.

The pre-post implementation comparison of ACIC domains and total score is displayed in Table 1. Pre-implementation ACIC total mean scores ranged from 3.70 in Vilnius to 7.90 in Andalusia. Post-implementation ACIC total mean scores varied from 5.52 in Vilnius to 8.04 in Kaunas. An increase in ACIC scores was also found across sites except in Andalusia, although they were not statistically significant in general (Figure 1). For the total sample, there was a significant increase in ACIC scores at the end of the intervention in Parts 3b to 4 and ACIC total. Effect sizes ranged from 0.58 (Part 1) to 1.10 (Part 4) for ACIC dimensions, and it was 0.83 for ACIC total scores.

### 3.2. PACIC+ Results

A convenience sample of 208 patients (52 from Andalusia, 61 from Rome, 50 from Kaunas, and 45 from Vilnius), consecutive in each site, completed the PACIC+ survey both pre- and post-implementation, and 18 patients from Aragon completed the PACIC+ post-implementation. The distribution by sites and their characteristics are displayed in Table 2. In general, women accounted for 52.2% of the total sample, who had a mean age of 62.9 (standard deviation, SD: 17.1; range: 20–93) years. The mean *change score* for the total sample was 4.91 (SD: 1.14); 58% of the total sample reported better care in the last 12 months.

The baseline PACIC+ summary score ranged from 2.91 (SD: 0.96) in Andalusia to 3.90 (SD: 0.78) in Vilnius (Table 3). *Arrange* was the domain with the lowest scores across sites, while *advise* had the highest scores. After the intervention, the PACIC+ summary score ranged from 3.46 (SD: 0.97) in Andalusia to 4.55 (SD: 0.35) in Aragon. As in baseline, *arrange* was the domain with the lowest scores in all sites and *advise* the domain with the highest scores.

**Figure 2:**
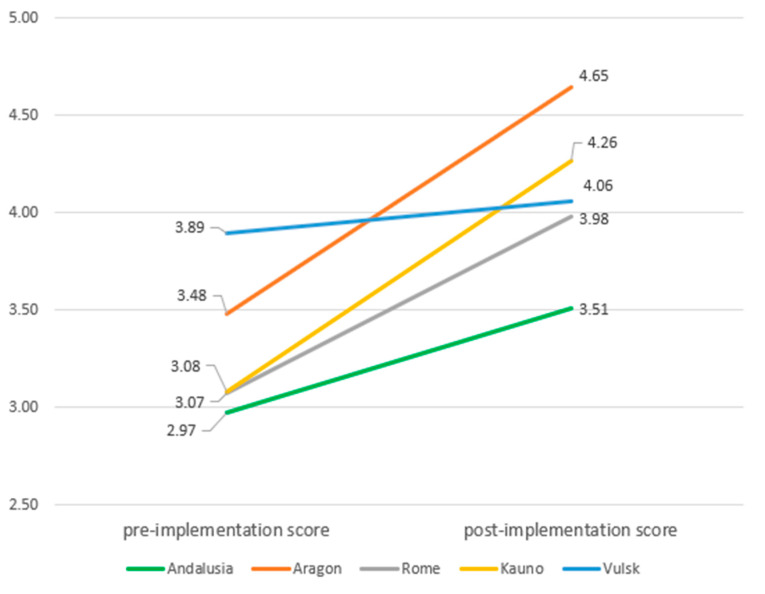
Estimated marginal means of PACIC+ 5As summary score by site and by pre- and post-implementation.

At follow-up, the sample was composed by 210 patients. A significant increase was found in the PACIC+ summary score (Table 3), ranging from 3.25 at baseline to 4.03 after the intervention (*p* < 0.001). PACIC+ domains also increased significantly, with *arrange* being the domain with the highest increase (0.99), although *advise* was the domain that reached the highest score (4.16, SD: 0.75). By sites, the lowest increases were observed in Vilnius (0.09 in *assist* to 0.26 in *arrange*) (Figure 2). The greatest changes were reported in Aragon (all domains except *assist*) and Kaunas (*assist*). Effect sizes ranged from 0.70 (*assist*) to 0.89 (*arrange*), with a value of 0.82 for the summary score.

The regression models of PACIC+ domains and summary score showed that a higher change in PACIC+ was mainly associated to lower scores in the corresponding PACIC+ domains at baseline (standardized beta, β = −0.72 in *advise*, to −0.692 in *assess*, *p* < 0.001) and to a higher change (β = 0.24 in *agree* to 0.34 in *assess*, *p* < 0.001) in ACIC domain 1 (delivery system organization) (Table 4). A higher change in PACIC+ domains *assess*, *assist,* and *arrange* and in summary score scores was consistently associated to a lower change in domain 4 of ACIC (integration). Changes in PACIC+ scores were not significantly associated to changes in ACIC domain 2, nor to socio-demographic characteristics. The explained variance (R^2^) ranged from 0.44 (PACIC+ *assess* model) to 0.55 (*advise*).

## 4. Discussion

The aim of this study was to assess the impact of the pilot implementation of the CHRODIS IMCM in five European sites considering the perspective of both healthcare managers and patients. Despite the existing differences among sites in terms of implemented components of the IMCM and target population, in general the IMCM had a positive effect across all healthcare systems in which it was tested. The total ACIC score increased from 5.23, indicative of basic support at baseline, to 6.71, which means reasonably good support for chronic illness care [19], representing a change of large magnitude (effect size = 0.83). Interventions also resulted in positive, statistically significant changes from the patients’ perspective in almost all sites.

An increase in scores in all ACIC domains was observed after the intervention, although it was only significant in parts related to decision support, delivery system design and clinical information systems, and integration of IMCM components. In our study, the *delivery system design* component was one of the dimensions that benefited most from the implementation of the IMCM, as indicated by its greater effect size. This component is present in most comprehensive care programs for multimorbidity [24]; however, addressing this dimension requires well-trained clinical teams that ensure successful self-management and coordinate preventive care [25]. In our study, all implementing sites developed evidence-base practices and a consultation system to support patient decision-making, linked to a clinical information system for providing clinicians with feedback and promoting continuity of care.

All sites except Andalusia showed an increase in ACIC total and domains scores, although in general not significant. The different non-significant trend observed in scores in Andalusia could be due to feeling of uncertainty by healthcare managers, consequent to political changes that occurred during the time of the intervention. However, to what degree ACIC scores can reflect variations in the political and economic context beyond the healthcare system is unknown.

According to the PACIC+ results, the interventions were generally well appreciated by patients. Their sociodemographic characteristics were similar to the population that attended each site. The magnitude of the change pre-post implementation was high, and greater than those reported by previous studies on guided care [26] or goal-setting interventions [27] for older adults with multimorbidity. Patients showed the lowest degree of satisfaction with care in the *arrange* dimension and the highest in the *advise* one. Despite the heterogeneity of samples in our intervention, these results are consistent with the ones found by previous studies [22,28,29], indicating the importance of improving actions to arrange follow-ups with patients.

The lower change reported by patients from one of the sites (Vilnius) might be due to a ceiling effect given that this was the site with the highest baseline PACIC+ scores, and therefore with the lowest room for improvement. The heavy users of healthcare resources targeted at the Vilnius pilot site were mainly patients with complex chronic conditions and a longer period of the intervention might be required to reach significant change in the quality of their care. Further studies will confirm that, due to the complexity of multimorbid patients’ cases, additional funding and new services for their care are needed to adapt to the emergent needs of the integrated care model.

About 50% of the variance in the change of PACIC+ scores was explained by baseline PACIC+ scores, as well as by baseline ACIC scores, independently of patients’ characteristics. The lack of association between patients´ characteristics and PACIC+ scores has been previously reported in some studies [22,30,31]. According to our findings, the higher change in patients’ satisfaction after the intervention was associated with a lower baseline value in the respective PACIC+ dimension, which could indicate that the least satisfied patients could benefit more from such interventions.

Higher patient satisfaction changes were consistently associated with a higher change in the ACIC delivery-system design dimension. The IMCM component on delivery system design was actually targeted in every site intervention, including the following aspects: regular comprehensive assessment of patients; multidisciplinary, coordinated teams; case managers; and individualized care plans [11]. The appointment of case managers had already resulted in a positive effect on the PACIC+ total score in heart-failure patients [32]. Results from both healthcare managers and patients concur in the importance of delivery system design, which should be targeted in public health policies aimed at improving care for patients with multimorbidity.

Several limitations in this study should be acknowledged. It included a patient-reported experience measure (PACIC+), but lacked a quality of life measure. However, previous interventions aimed at improving care for patients with multimorbidity have not changed their quality of life. Instead, an improvement in patient satisfaction is observed, which can be considered in itself a relevant positive outcome [5,32]. Far from being a controlled randomized clinical trial, the methodological design of the study involved the collection of what can be called “real world data”, which implies some constraints, such as non-representative samples, missing data for some measures (i.e., change score in Italy due to administrative difficulties), smaller sample size or lack of PACIC+ baseline data in Aragon. This led to the need of imputing baseline scores, and more studies are needed to confirm the validity of the results. Each site set its own inclusion criteria, and thus, participants were heterogeneous in their sociodemographic and clinical characteristics. The interventions had a common structured methodology and duration but varied in type of setting and healthcare levels. Although this could be considered a limitation, it represented an opportunity to test the IMCM in a variety of situations and to show that the IMCM can be adapted to the characteristics of different healthcare systems. Moreover, despite the differences between sites, components of the five IMCM domains have been implemented and assessed. Finally, a longer follow-up would be desirable to detect changes that could occur in the long term.

Despite its limitations, this study provides intervention results on the pilot application of the IMCM in five European settings of both primary and specialized care levels, with different characteristics. Results consistently showed an improvement in the quality of care from the perspective of patients and healthcare managers. Our results underscore the benefits of a comprehensive approach to multimorbidity care, highlighting the need to integrate the IMCM in National Health Systems to lessen the burden that multimorbidity represents for healthcare managers, stakeholders, and patients.

## Figures and Tables

**Table 1:** Mean scores of the Assessment of Chronic Illness Care (ACIC) survey subscales and total scale, before (pre) and after (post) implementation of the Integrated Multimorbidity Care Model (IMCM), by site.

		Andalusia			Aragon			Rome			Kaunas			Vilnius			Total			
		Mean	SD	*p*	Mean	SD	*p*	Mean	SD	*p*	Mean	SD	*p*	Mean	SD	*p*	Mean	SD	*p*	ES
**Part 1**	**Pre**	10.42	0.59		6.39	1.89		8.33	1.41		6.58	0.35		5.01	0.87		6.91	2.16		
	**Post**	8.40	1.46		8.44	1.51		8.92	0.59		8.92	0.35		6.88	1.42		8.16	1.39		
	**Diff**	−2.02		0.130	2.05		0.216	0.59		0.644	2.34		0.022	1.87		0.066	1.25	1.65	0.072	0.58
**Part 2**	**Pre**	8.17	0.24		5.11	1.68		5.33	0.47		5.50	1.18		4.00	1.25		5.33	1.72		
	**Post**	7.40	2.16		7.11	1.83		6.50	2.12		7.50	0.24		4.75	0.63		6.58	1.84		
	**Diff**	−0.77		0.657	2.00		0.236	1.17		0.527	2.00		0.143	0.75		0.324	1.25	0.86	0.071	0.72
**Part 3a**	**Pre**	7.62	0.88		6.42	2.31		5.12	3.00		5.12	0.88		3.94	1.16		5.44	1.98		
	**Post**	6.60	1.72		7.92	1.91		6.75	3.18		8.00	0.35		4.88	1.76		6.61	2.00		
	**Diff**	−1.02		0.475	1.5		0.435	1.63		0.652	2.88		0.051	0.94		0.408	1.17	1.35	0.128	0.59
**Part 3b**	**Pre**	7.50	1.06		4.75	2.54		4.13	2.30		5.38	0.88		3.06	0.43		4.65	2.01		
	**Post**	5.90	2.07		6.89	1.05		4.50	2.83		8.88	1.18		6.13	1.45		6.34	1.92		
	**Diff**	−1.60		0.362	2.14		0.249	0.37		0.898	3.50		0.032	3.07		0.007	1.69	1.21	0.026	0.84
**Part 3c**	**Pre**	8.17	0.71		6.72	2.04		5.42	0.59		6.08	0.59		4.17	0.76		5.86	1.73		
	**Post**	6.73	1.30		8.50	1.20		7.67	0.71		9.50	0.47		6.13	1.74		7.38	1.65		
	**Diff**	−1.44		0.216	1.78		0.263	2.25		0.074	3.42		0.024	1.96		0.085	1.52	1.74	0.026	0.88
**Part 3d**	**Pre**	7.40	1.41		6.20	3.12		4.20	1.41		3.50	1.27		3.00	0.54		4.68	2.28		
	**Post**	6.84	0.92		7.67	2.68		5.40	0.85		6.90	1.27		4.90	1.99		6.34	1.81		
	**Diff**	−0.56		0.548	1.47		0.571	1.20		0.412	3.40		0.116	1.90		0.115	1.66	3.32	0.035	0.73
**Part 4**	**Pre**	6.00	0.47		3.94	2.42		2.92	1.30		4.08	0.35		2.71	0.77		3.74	1.63		
	**Post**	5.50	1.60		6.11	1.84		4.92	0.59		6.58	1.06		4.96	1.81		5.54	1.50		
	**Diff**	−0.50		0.697	2.17		0.285	2.00		0.185	2.5		0.087	2.25		0.063	1.8	0.976	0.011	1.10
**Total**	**Pre**	7.90	0.29		5.65	2.20		5.06	1.50		5.18	0.43		3.70	0.72		5.23	1.78		
	**Post**	6.77	1.39		7.52	1.64		6.38	1.35		8.04	0.14		5.52	1.30		6.71	1.45		
	**Diff**	−1.13		0.330	1.87		0.303	1.32		0.454	2.86		0.012	1.82		0.051	1.48	1.41	0.022	0.83

Mann-Whitney test. Pre: Pre-implementation score; Post: Post-implementation score; Diff: Difference in scores post-pre implementation; SD: Standard deviation; ES: Effect size; ACIC components: Part 1: Delivery system organization; Part 2: Community linkages; Part 3a: Self-management support; Part 3b: Decision support; Part 3c: Delivery system design; Part 3d: Clinical information systems; and Part 4: Integration of IMCM components.

**Table 2:** Socio-demographic characteristics of patients and self-reported change score by the implementing site.

		Andalusia (n = 52)		Aragon (n = 18)		Rome (n = 61)		Kaunas (n = 50)		Vilnius (n = 45)		Total (n = 226)	
		n	%	n	%	n	%	n	%	n	%	n	%
**Sex**	**Man**	25	48.1	10	55.6	33	54.1	18	36.0	22	48.9	108	47.8
	**Woman**	27	51.9	8	44.4	28	45.9	32	64.0	23	51.1	118	52.2
**Civil status**	**Single**	3	5.77	0	0.00	48	78.7	1	2.0	2	4.44	54	23.9
	**Married/with partner**	34	65.4	12	66.7	11	18.0	24	48.0	35	77.8	116	51.3
	**Widowed**	13	25.0	5	27.8	0	0.00	13	26.0	3	6.7	34	15.0
	**Separated/divorced**	2	3.85	1	5.56	2	3.28	12	24.0	5	11.1	22	9.73
**Education**	**Primary complete or incomplete**	30	57.7	15	83.3	22	36.1	0	0.0	0	0.0	67	29.7
	**Secondary**	12	23.1	1	5.56	30	49.2	22	44.0	6	13.3	71	31.4
	**University**	5	9.62	2	11.1	3	4.92	28	56.0	29	64.4	67	29.7
	**Other**	5	9.62	0	0.00	6	9.84	0	0.0	10	22.2	21	9.3
**Activity**	**Employee**	4	7.69	0	0.00	9	14.8	13	26.0	22	48.9	48	21.2
	**Housewife**	11	21.2	1	5.56	3	4.92	0	0.0	1	2.2	16	7.1
	**Unemployed**	3	5.77	0	0.00	16	26.2	0	0.0	1	2.2	20	8.85
	**Retired**	30	57.7	17	94.4	21	34.4	34	68.0	20	44.4	122	54.0
	**Other**	4	7.69	0	0.00	12	19.7	3	6.0	1	2.2	20	8.85
**Change score**	**No change or worse (1–4)**	11	26.2	4	22.2	--	--	31	36.0	12	42.9	58	42.0
	**Better (5–7)**	31	73.8	14	77.8	--	--	19	64.0	16	57.1	80	58.0
		**M**	**SD**	**M**	**SD**	**M**	**SD**	**M**	**SD**	**M**	**SD**	**M**	**SD**
**Age (years)**		72.3	12.9	80.1	9.5	46.7	19.4	68.5	6.4	61.0	9.1	62.9	17.1
**Change score (1–7)**		5.31	1.14	5.8	1.2	--	--	4.4	1.0	4.7	0.8	4.9	1.1

M: Mean; SD: Standard deviation.

**Table 3:** Mean scores of Patient Assessment of Care for Chronic Conditions (PACIC+) survey subscales and total scale, before (pre) and after (post) implementation, by site.

		Andalusia (n = 42)			Aragon (n = 18)			Rome (n = 60)			Kaunas (n = 50)			Vilnius (n = 40)			Total (n = 210)			
		Mean	SD	*p*	Mean	SD	*p*	Mean	SD	*p*	Mean	SD	*p*	Mean	SD	*p*	Mean	SD	*p*	Effect Size
**Assess**	**Pre**	2.96	1.14		3.45	0.35		3.28	1.11		3.20	0.93		3.95	0.84		3.34	1.03		
	**Post**	3.56	1.10		4.58	0.39		4.09	0.76		4.44	0.75		4.05	0.83		4.10	0.88		
	**Diff**	0.60	0.87	<0.001	1.13	0.39	<0.001	2.02	1.50	<0.001	1.24	0.98	<0.001	0.10	0.54	0.226	0.77	0.96	<0.001	0.75
**Advise**	**Pre**	3.20	0.91		3.20	1.07		3.28	1.10		3.24	0.95		4.10	0.73		3.42	0.99		
	**Post**	3.69	0.88		4.60	0.48		4.14	0.66		4.36	0.68		4.23	0.70		4.16	0.75		
	**Diff**	0.49	1.04	0.004	1.21	0.73	<0.001	0.87	1.05	<0.001	1.12	0.94	<0.001	0.14	0.62	0.177	0.74	0.97	<0.001	0.75
**Agree**	**Pre**	3.00	1.02		3.41	0.61		3.18	1.06		2.90	1.18		3.87	0.90		3.23	1.07		
	**Post**	3.54	1.10		4.53	0.39		4.07	0.68		4.09	0.93		4.09	0.77		4.01	0.87		
	**Diff**	0.53	0.93	0.001	1.13	0.49	<0.001	0.82	1.11	<0.001	1.18	1.24	<0.001	0.21	0.73	0.072	0.78	1.01	<0.001	0.73
**Assist**	**Pre**	2.48	1.03		2.81	0.68		2.93	1.14		3.20	0.86		3.78	1.04		3.05	1.08		
	**Post**	3.10	1.02		3.98	0.76		3.94	0.76		4.17	0.76		3.87	0.82		3.82	0.90		
	**Diff**	0.62	0.92	<0.001	1.16	0.67	<0.001	0.54	1.25	<0.001	0.97	0.93	<0.001	0.09	0.79	0.475	0.76	0.96	<0.001	0.70
**Arrange**	**Pre**	2.03	1.01		2.20	0.57		2.62	1.15		2.26	1.07		3.17	1.09		2.48	1.11		
	**Post**	2.70	1.05		3.63	0.94		3.82	0.89		3.71	0.98		3.43	0.88		3.48	1.03		
	**left**	0.66	0.98	<0.001	1.44	0.60	<0.001	0.93	1.29	<0.001	1.45	1.40	<0.001	0.26	0.77	0.040	0.99	1.19	<0.001	0.89
**5As Summary**	**Pre**	2.91	0.96		3.38	0.54		3.17	1.01		3.05	0.87		3.90	0.78		3.25	0.95		
	**Post**	3.46	0.97		4.55	0.35		4.07	0.66		4.23	0.81		4.07	0.75		4.03	0.82		
	**Diff**	0.54	0.85	<0.001	1.17	0.58	<0.001	0.72	1.05	<0.001	1.19	0.93	<0.001	0.17	0.62	0.093	0.78	0.90	<0.001	0.82

Paired Student’s *t*-test; Diff: Difference in scores post-pre implementation; SD: Standard deviation; Missing data on PACIC+ pre, for Aragon: Imputed with the regression model.

**Table 4:** Linear regression models of Patient Assessment of Care for Chronic Conditions (PACIC+) domains at post-implementation.

Model		Standardized Beta	t	Sig.	95% Confidence Interval		R^2^
**Assess**	***(Constant)***		*6.085*	*<0.001*	*1.665*	*3.261*	**0.44**
	**Assess pre**	−0.604	−10.849	<0.001	−0.667	−0.462	
	**ACIC1 diff.**	0.335	4.220	<0.001	0.107	0.296	
	**ACIC4 diff.**	−0.212	−2.767	0.006	−0.359	−0.060	
**Advise**	***(Constant)***		*8.925*	*<0.001*	*2.488*	*3.899*	**0.55**
	**ACIC1 diff.**	0.266	3.742	<0.001	0.077	0.248	
	**Advise pre**	−0.716	−14.543	<0.001	−0.803	−0.611	
**Agree**	***(Constant)***		*7.247*	*<0.001*	*2.015*	*3.522*	**0.48**
	**ACIC1 diff.**	0.243	3.172	0.002	0.058	0.250	
	**Agree pre**	−0.658	−12.304	<0.001	−0.717	−0.519	
**Assist**	***(Constant)***		*6.940*	*<0.001*	*1.844*	*3.307*	**0.46**
	**ACIC1 diff.**	0.296	3.737	<0.001	0.085	0.273	
	**ACIC4 diff.**	−0.225	−3.050	0.003	−0.369	−0.079	
	**Assist pre**	−0.656	−11.762	<0.001	−0.684	−0.488	
**Arrange**	***(Constant)***		*6.553*	*<0.001*	*2.023*	*3.765*	**0.46**
	**ACIC1 diff.**	0.287	3.679	<0.001	0.099	0.328	
	**ACIC4 diff.**	−0.242	−3.283	0.001	−0.476	−0.119	
	**Arrange pre**	−0.649	−11.836	<0.001	−0.806	−0.576	
**5As summary**	***(Constant)***		*6.867*	*<0.001*	*1.802*	*3.254*	**0.45**
	**ACIC1 diff.**	0.299	3.812	<0.001	0.082	0.258	
	**ACIC4 diff.**	−0.178	−2.333	0.021	−0.306	−0.026	
	**5As summary pre**	−0.625	−11.270	<0.001	−0.697	−0.490	

Other variables included in the models: Sex, age, civil status, education, activity, ACIC2 diff. Pre: Pre-implementation score. Diff.: Difference in scores between post- and pre-implementation.
